# Alcohol use in the first three years of bereavement: a national representative survey

**DOI:** 10.1186/1747-597X-7-3

**Published:** 2012-01-16

**Authors:** János Pilling, Barna Konkolÿ Thege, Zsolt Demetrovics, Mária S Kopp

**Affiliations:** 1 Institute of Behavioral Sciences, Semmelweis University; Nagyvárad tér 4., Budapest, 1089. Hungary; 2 Institute of Psychology, Eötvös Loránd University; Izabella u. 46., Budapest, 1064. Hungary

**Keywords:** bereavement, alcohol consumption, national representative survey, gender differences

## Abstract

**Background:**

Earlier results concerning alcohol consumption of bereaved persons are contradictory. The aim of the present study was to analyze the relationship between bereavement and alcohol consumption accounting for time and gender differences on a nationally representative sample from Hungary ("Hungarostudy Epidemiological Panel Survey", N = 4457)

**Methods:**

Drinking characteristics of mourning persons (alcohol consumption, dependence symptoms, and harmful consequences of alcohol use) in the first three years of grief were examined among persons between 18-75 years using the Alcohol Use Disorders Identification Test (AUDIT).

**Results:**

Men bereaved for one year scored higher on two dimensions of AUDIT (dependence symptoms and harmful alcohol use), while men bereaved for two years scored higher on all three dimensions of AUDIT compared to the non-bereaved. The rate of men clinically at-risk concerning alcohol consumption among the non-bereaved is 12.9%, and among men bereaved for one year is 18.4% (a non-significant difference), while 29.8% (p < 0.001, OR = 2,781) among men bereaved for two years. However, men bereaved for three years did not differ from the non-bereaved in their drinking habits. In case of bereaved women, again no difference was found with respect to alcohol use compared to the non-bereaved.

**Conclusion:**

Among bereaved men, the risk of alcohol related problems tends to be higher, which can be shown both among men bereaved for one year as well as men bereaved for two years. Considering the higher morbidity and mortality rates of bereaved men, alcohol consumption might play a mediator role. These facts draw attention to the importance of prevention, early recognition, and effective therapy of hazardous drinking in bereaved men.

## Introduction

Several studies have confirmed that death of a close relative is a serious emotional burden, which increases the frequency of different physical and mental disorders [1-3]. Studies have also pointed out that specific stressful life events (divorce, financial difficulties, etc.) lead to increased alcohol consumption [4]. Since grief involves severe stress [5,1], based on the tension reduction hypothesis [6], an increase in the frequency and amount of alcohol consumption would be expected during this period. However, the results of these studies are rather dubious. Although some studies indeed indicated an increased frequency of alcohol consumption and alcohol dependence among grieving people [7,8], other studies did not find clear differences between grieving people and non-grieving individuals regarding the extent of alcohol related problems [9,10].

Although researchers agree that bereavement can significantly affect people's physical and mental health status, the duration of this effect is also debated. The majority of studies indicate that grief influences health in the earlier phase of bereavement, but usually these effects disappear in one year's time [11,12]. At the same time, other studies suggest that grief might even influence long term health status [13,2]. Furthermore, according to some studies on the drinking habits of grieving people, bereavement increases alcohol consumption only for a short period [7]. On the other hand, some studies report elevated alcohol use even after several years [9]. One possible reason for the inconsistent results is that not all previous studies had considered the relevance of gender differences, although these differences had been well documented both in the aspect of the grief reaction and psychoactive substance use: widowers are indeed relatively more vulnerable than widows, and men have more drinking problems than women [14,15,5]. In the background of gender differences regarding grief reactions we may discover different ways of expressing emotion as well as a diversity of coping styles, not to mention the different levels of socialization and those of social support [14].

In connection with the findings above, the aim of the present study was to analyze the relationship between bereavement and alcohol consumption accounting for time and gender differences on a national (Hungarian) representative sample in order to avoid any bias resulting from the method of data collection.

## Methods

### Participants

The present analyses were based on cross-sectional data from the Hungarostudy Epidemiological Panel Survey (HEP) 2006, a follow-up of the Hungarostudy 2002 nation-wide representative survey. The sample was drawn from the National Population Register. The baseline data collection took place in 2002 and involved 12,668 subjects, who were representative of the adult population of Hungary according to age, gender, and 150 sub-regions. Those participants of the study, who had given consent for the follow-up, were contacted by our interviewers once again in 2006. Not counting those who had died, rejected to answer or were not able to answer the questions (e.g. due to their illness), finally, 4457 persons filled out the questionnaire in 2006. Regarding gender, age, and regions, the sample of persons-following the weighing of the data-proved to be representative of the adult (18-75 years) Hungarian population. The sampling methods are described in detail elsewhere [16]. The study was approved by the Ethics Committee of the Semmelweis University in Budapest.

Our study included 466 persons from the sample of the HEP 2006 survey, who had lost a close relative (spouse, mother, or father) in the past three years. Regarding the period of bereavement, the following subsamples were created: loss in one year comprising 181 persons (76 males, 105 females), loss in two years comprising 127 persons (57 males, 70 females, and loss in three years comprising 158 persons (60 males, 98 females). Overall, 85.7% of the bereaved lost their mother or father while 14.3% of them lost their spouse. There were no significant differences between the total sample and the study sample concerning gender, age, and education. Demographic characteristics of the sample are shown in Table 1.

**Table 1:** Demographic characteristics of the study sample

	Bereaved for one year	Bereaved for two years	Bereaved for three years	Non- bereaved	Total sample
N	181	127	158	3991	4457
gender (% of females)	58,0	55,1	62,0	59,9	59,5

Age					
- males					
mean	47,2	47,5	45,6	46,0	46,4
SD	(14,11)	(11,47)	(13,90)	(17,14)	(16,65)
- females					
mean	49,9	55,3	48,1	49,8	49,9
SD	(13,95)	(13,02)	(13,45)	(18,59)	(17,94)

Education					
- males					
elementary	23.1%	16.8%	12.3%	18.8%	18.8%
secondary	66.9%	68.9%	66.2%	67.7%	67.6%
higher ed.	10.0%	14.3%	21.5%	13.5%	13.5%
	
- females					
elementary	35.2%	32.6%	24.8%	32.8%	32.8%
secondary	56.0%	55.1%	65.2%	51.5%	52.4%
higher ed.	8.8%	12.2%	10.0%	15.6%	14.8%

### Measures

The HEP 2006 survey used a slightly modified Hungarian version [17] of the Alcohol Use Disorders Identification Test (AUDIT) [18] to assess the risk of alcohol use. (While the second item of the original questionnaire inquired after the total amount of alcohol consumed daily, the version we used contained more detailed answer options referring to the consumed amounts of specific types of drinks-beer, wine, spirits-that were converted to the original answer categories for the analysis.) The questionnaire, which has excellent reliability and validity characteristics, has been frequently used to estimate alcohol related problems worldwide [19]. AUDIT has a maximum score of 40. In line with international routine, those persons were considered at-risk who scored 8 or higher on the questionnaire. The questionnaire consists of 10 items that are assigned to three domains: hazardous alcohol use (1-3 items), dependence symptoms (4-6), and harmful consequences of alcohol use (7-10). Following the calculation of total scores, the domains of the questionnaire were analyzed separately. During this analysis, we calculated with the original scores (0-4) of the questionnaire in case of the first domain, while in cases of the second and third domains the answer categories were dichotomized (due to the low number of elements in each category). To the "yes" category belonged all those who reported the presence of dependence symptoms and harmful consequences of alcohol use.

### Statistical analysis

The statistical analysis was executed with the SPSS 15.0 software. The ordinal regression model was used to analyze the data. Analyses were carried out separately for both genders, and the results were adjusted for age and education, since, according to former studies, these factors may influence alcohol use.

## Results

With regard to the total score of AUDIT, the T-Test significant indicated gender differences in our sample (p < .001, t = 6.501), which confirmed the rationale behind analyses carried out in gender breakdown. In case of men bereaved for one year, two dimensions of the AUDIT (dependence symptoms: OR = 2.07, p < 0.005 and harmful alcohol use: OR = 2.64, p < 0.001), while in case of men who were bereaved for two years all three dimensions were higher (hazardous alcohol use: OR = 1.94, p < .001, dependence symptoms: OR = 3.45, p < 0.001, harmful alcohol use: OR = 2.45, p < 0.05) compared to the non-bereaved. The rate of men clinically at-risk concerning alcohol use was 12.9% among the non-bereaved, and 18.4% among men bereaved for one year (the difference is insignificant), while it was 29.8% among men bereaved for two years (p < 0.001, OR = 2.781). However, among men bereaved for three years, no significant differences regarding alcohol consumption were found, when compared to non-bereaved men. Among women we have not found significant differences regarding any aspects of alcohol consumption in the first three years of bereavement. The AUDIT total score was significantly high only among men bereaved for two years (p < 0.001, OR = 2.783). (Table 2). Concerning the AUDIT total score, we also tested the possible interaction between gender and bereavement status. Analyses carried out with ordinal regression have confirmed that, regarding the Audit total score, gender differences can only be pointed out among persons bereaved only for two years (p < 0.01, Wald chi square: 6.651)

**Table 2:** Alcohol consumption of persons in bereavement

	Bereaved for one year		Bereaved for two years		Bereaved for three years		Non-bereaved-reference category	
	
	Male	Female	Male	Female	Male	Female	Male	Female
**AUDIT total score**								
Mean	4.83	1.29	6.59	0.96	3.72	1.01	3.99	1.11
SD	4.37	2.24	6.95	1.59	3.60	1.34	4.13	1.99
At-risk	18,4%	2,8%	29,8%	1,4%	11,7%	1,0%	12,9%	2,0%
Wald chi-squares	1.433	0.598	11.450	0.347	0.032	0.338		
Odds Ratio	1.445	1.569	2,783***	0.464	0.930	0.583		

**Hazardous alcohol use**								
Mean	4.88	1.47	5.63	1.24	4.32	1.33	4.45	1.29
SD	3.25	2.02	3.85	2.06	3.34	1.65	3.40	1.95
Wald chi-squares	1.316	1.348	7,871	< 0.001	0.112	1.074		
Odds Ratio	1.27	1.25	1.94**	1.0	0.93	1.23		

**Dependence symptoms**								
Mean	0.24	0.00	0.30	0.00	0.01	0.00	0.10	0.01
SD	0.68	0.00	0.76	0.00	0.11	0.00	0.42	0.15
Wald chi-squares	4.075	< 0.001	11.075	< 0.001	1.811	< 0.001		
Odds Ratio	2.07*	1.00	3.45***	1.00	0.19	1.00		

**Harmful alcohol use**								
Mean	0.39	0.00	0.40	0.01	0.05	0.01	0.14	0.02
SD	0.86	0.00	1.01	0.09	0.23	0.11	0.50	0.20
Wald chi-squares	10.668	< 0.001	6.385	0.111	0.892	0.072		
Odds Ratio	2.64***	1.00	2.45*	0.63	0.57	0.78		

Ordinal regression model *: p < 0.05, **: p < 0.01, ***: p < 0.001, df = 1. (Adjusted for age and education).

## Discussion

The results of our study, carried out on a national representative sample from Hungary, confirm the results of previous smaller sample studies, which indicated an elevated risk of alcohol related problems among bereaved persons [7,8]. Our results, nevertheless, also indicate gender differences in alcohol consumption during the period of bereavement. While the results indicated a significantly greater frequency of alcohol related problems in case of men, these alcohol related disorders did not afflict women. A few earlier studies had indicated similar gender differences [20,4], while other studies showed that hazardous alcohol use was more common in depressed men than women [21,22]. On the other hand, a study of Dawson and his colleagues [4], carried out on a sample of 26946 persons, showed an unambiguous positive association between the number of stressors in the previous year and heavy drinking. It seems plausible that the relationship between harmful drinking and bereavement in men is mediated by depression and anxiety, which deserves further examination.

Our results have been controlled for age and gender because, according to former studies, these may influence alcohol use. At the same time, many other factors might have an influence on alcohol use as well (e.g. anxiety, social support, spirituality) and the examination of their roles requires further research.

Relatively few studies highlight and address the long-term effects of bereavement, that is, the effects that last for more than one year [3,13]. The results of our study, on the other hand, indicate that the effects of stress associated with bereavement might exceed the one- year duration ascertained by most of the previous studies. Our study, however, did not show differences among men bereaved for three years, which possibly indicates that coping with the effects of losing someone takes place primarily in the first two years of bereavement.

The strength of our study is that it included a national, representative sample, providing the opportunity to analyze the long-term (3 years) effects of bereavement. Moreover, it considers several components of alcohol consumption (amount of alcohol consumed, frequency of drinking, alcohol dependence, and harmful effects of alcohol use). The limitation of the study, nevertheless, is its cross-sectional nature, thus it cannot verify causal relationships. Since in our study numerous interrelated factors have been analyzed, results can be filtered by applying the method of the Bonferroni correction (0.05/24). Accordingly, the significant results concerning harmful alcohol use in persons bereaved for one year, the dependence symptoms and the total score of AUDIT among persons bereaved for two years indicate higher vulnerability of alcohol misuse, thus results do not change considerably after the correction, either. A further limitation of the study is the self-rating method applied, which means that responses may not be altogether objective. When interpreting the results, we also have to consider that women might perform differently on tests that require the estimation of alcohol consumption, and might be more likely to hide their alcohol related problems [23].

Several large sample or review studies confirm that in the period of bereavement, morbidity and mortality data increase to a greater extent for men than for women [1,3]. Considering these facts, our study points out that excessive alcohol consumption can be an important factor. Although alcohol use is a maladaptive way of coping, in spite of its risks it is one of the traditional forms of reducing stress in western society. Women express their feelings more overtly than men (14), therefore men are more at risk to resort to drink in order to relieve stress. Because bereaved persons more often ask for medical help [1], health care professionals-especially GPs-could be the ones to recognize bereavement related alcohol problems in time. Helping people to cope with their loss is crucial both in treatment and prevention, and in this process, besides professionals who get in touch with bereaved persons, self help groups also have an important role. At the same time, it is a special difficulty that persons with alcohol related problems are often reluctant to seek face to face professional help, and it follows that useful information available online, as well as anonymous helpline and developing telemedicine services play an increasingly important role in helping bereaved men [24]. The results of our study highlight that more effective help offered for bereaved men may contribute to reducing alcohol related health problems and alcohol related mortality.

## Competing interests

The authors declare that they have no competing interests.

## Authors' contributions

JP designed the study and (cooperation with ZsD) managed the literature searches and analyses. BKT and ZsD conducted the statistical analysis. MK designed and managed the HEP 2006 research. JP wrote the first draft of the manuscript. All authors read and approved the final manuscript.

## References

[B1] Stroebe M Schut H Stroebe W Health outcomes of bereavement Lancet 2007 370 1960 1973 10.1016/S0140-6736(07)61816-9 18068517

[B2] Li J Precht DH Mortensen PB Olsen J Mortality in parents after death of a child in Denmark: A nationwide follow-up study Lancet 2003 361 363 367 10.1016/S0140-6736(03)12387-2 12573371

[B3] Martikainen P Valkonen T Mortality after the death of a spouse: Rates and causes of death in a large Finnish cohort Am J Public Health 1996 8 1087 1093 10.2105/ajph.86.8_pt_1.1087 PMC1380614 8712266

[B4] Dawson DA Grant BF Ruan WJ The association between stress and drinking: Modifying effects of gender and vulnerability Alcohol and Alcoholism 2005 5 453 460 10.1093/alcalc/agh176 15972275

[B5] Chen JH Bierhals AJ Prigerson HG Kasl SV Mazure CM Jacobs S Gender differences in the effects of bereavement-related psychological distress in health outcomes Psychol Med 1999 29 367 380 10.1017/S0033291798008137 10218927

[B6] Conger JJ Alcoholism: Theory, problem and challenge. II. Reinforcement theory and the dynamics of alcoholism Q J Studies Alcohol 1956 17 296 305 13336262

[B7] Perreira KM Sloan FA Life events and alcohol consumption among mature adults: A longitudinal analysis J Stud Alc 2001 62 501 508 10.15288/jsa.2001.62.501 11513228

[B8] Byrne GJ Raphael B Arnold E Alcohol consumption and psychological distress in recently widowed older men Aust N Z J Psych 1999 5 740 747 10.1080/j.1440-1614.1999.00614.x 10545000

[B9] Pfefferbaum B Doughty DE Increased alcohol use in a treatment sample of Oklahoma City bombing victims Psychiatry 2001 4 296 303 10.1521/psyc.64.4.296.18598 11822207

[B10] Lee S Cho E Grodstein F Kawaci I Hu FB Colditz GA Effects of marital transitions on changes in dietary and other health behaviours in US women Int J Epidemiol 2005 34 69 78 15231759 10.1093/ije/dyh258

[B11] Manor O Eisenbach Z Mortality after spousal loss: Are there socio-demographic differences? Soc Sci Med 2003 56 405 413 10.1016/S0277-9536(02)00046-1 12473324

[B12] Lichtenstein P Gatz M Berg S A twin study of mortality after spousal bereavement Psychol Med 1998 28 635 643 10.1017/S0033291798006692 9626719

[B13] Johnson NJ Backlund E Sorlie PD Loveless CA Marital status and mortality: The National Longitudinal Mortality Study Ann Epidemiol 2000 4 224 238 10.1016/s1047-2797(99)00052-6 10854957

[B14] Stroebe M Stroebe W Schut H Gender differences in adjustment to bereavement: An empirical and theoretical review Rev Gen Psych 2001 1 62 83

[B15] Hanna EZ Grant BF Gender differences in DSM-IV alcohol use disorders and major depression as distributed in the general population: Clinical implications Compr Psychiatry 1997 4 202 212 10.1016/s0010-440x(97)90028-6 9202877

[B16] Susánszky É Székely A Szabó G Szántó Zs Klinger A Konkolÿ Thege B Kopp M A Hungarostudy Egészség Panel (HEP) felmérés módszertani leírása. [Methodological description of the Hungarian epidemiological panel (HEP) survey] Mentálhigiéné és Pszichoszomatika 2007 8 259 276

[B17] Gerevich J Bácskai E Rózsa S A kockázatos alkoholfogyasztás prevalenciája Psych Hung 2006 1 45 56 16783031

[B18] Saunders J B Aasland OG Babor TF De La Fuente JR Grant M Development of the Alcohol Use Disorders Identification Test (AUDIT): WHO collaborative project on early detection of persons with harmful alcohol consumption-II Addiction 1993 88 791 804 10.1111/j.1360-0443.1993.tb02093.x 8329970

[B19] Reinert DF Allen JP The alcohol use disorders identification test (AUDIT): A review of recent research Alcohol Clin Exp Res 2002 2 272 279 11964568

[B20] Buckley T Bartrop R McKinley S Ward C Bramwell M Roche D Mihailidou AS Morel-Kopp MC Spinaze M Hocking B Goldston K Tennant C Tofler G Prospective study of early bereavement on psychological and behavioural cardiac risk factors Int Med J 2009 39 370 378 10.1111/j.1445-5994.2008.01879.x 19460057

[B21] Kessler RC Crum RM Warner LA Nelson CB Schulenberg J Anthony JC Lifetime co-occurrence of DSM III-R alcohol abuse and dependence with other psychiatric disorders in the National Comorbidity Survey Arch Gen Psychiatry 1997 51 313 321 10.1001/archpsyc.1997.01830160031005 9107147

[B22] Marcus SM Young EA Kerber KB Kornstein S Farabaugh AH Mitchell J Wisniewski SR Balasubramani GK Trivedi MH Rush AJ Gender differences in depression: Findings from the STAR*D study J Affect Disord 2005 2-3 141 150 10.1016/j.jad.2004.09.008 15982748

[B23] Bradley KA Boyd-Wickizer J Powell SH Burman ML Alcohol screening questionnaires in women: a critical review JAMA 1998 2 166 171 10.1001/jama.280.2.166 9669791

[B24] White A Kavanagh D Stellman H Klein B Kay-Lambkin F Proudfoot J Drennan J Connor J Baker A Hines E Young R Online alcohol interventions: a systematic review J Med Internet Res 2010 12 5 e62 21169175 10.2196/jmir.1479 PMC3057310

